# Performance of a subsidised mammographic screening programme in Malaysia, a middle-income Asian country

**DOI:** 10.1186/s12889-017-4015-3

**Published:** 2017-01-28

**Authors:** Marianne Lee, Shivaani Mariapun, Nadia Rajaram, Soo-Hwang Teo, Cheng-Har Yip

**Affiliations:** 1 Cancer Research Malaysia, Subang Jaya, Malaysia; 2 University of Malaya, Kuala Lumpur, Malaysia; 3 Subang Jaya Medical Centre, No 1, Jalan SS12/1A, 47500 Subang Jaya, Malaysia

**Keywords:** Opportunistic mammographic screening, Breast cancer, Asia

## Abstract

**Background:**

The incidence of breast cancer in Asia is increasing because of urbanization and lifestyle changes. In the developing countries in Asia, women present at late stages, and mortality is high. Mammographic screening is the only evidence-based screening modality that reduces breast cancer mortality. To date, only opportunistic screening is offered in the majority of Asian countries because of the lack of justification and funding. Nevertheless, there have been few reports on the effectiveness of such programmes. In this study, we describe the cancer detection rate and challenges experienced in an opportunistic mammographic screening programme in Malaysia.

**Methods:**

From October 2011 to June 2015, 1,778 asymptomatic women, aged 40–74 years, underwent subsidised mammographic screening. All patients had a clinical breast examination before mammographic screening, and women with mammographic abnormalities were referred to a surgeon. The cancer detection rate and variables associated with a recommendation for adjunct ultrasonography were determined.

**Results:**

The mean age for screening was 50.8 years and seven cancers (0.39%) were detected. The detection rate was 0.64% in women aged 50 years and above, and 0.12% in women below 50 years old. Adjunct ultrasonography was recommended in 30.7% of women, and was significantly associated with age, menopausal status, mammographic density and radiologist’s experience. The main reasons cited for recommendation of an adjunct ultrasound was dense breasts and mammographic abnormalities.

**Discussion:**

The cancer detection rate is similar to population-based screening mammography programmes in high-income Asian countries. Unlike population-based screening programmes in Caucasian populations where the adjunct ultrasonography rate is 2–4%, we report that 3 out of 10 women attending screening mammography were recommended for adjunct ultrasonography. This could be because Asian women attending screening are likely premenopausal and hence have denser breasts. Radiologists who reported more than 360 mammograms were more confident in reporting a mammogram as normal without adjunct ultrasonography compared to those who reported less than 180 mammograms.

**Conclusion:**

Our subsidised opportunistic mammographic screening programme is able to provide equivalent cancer detection rates but the high recall for adjunct ultrasonography would make screening less cost-effective.

**Electronic supplementary material:**

The online version of this article (doi:10.1186/s12889-017-4015-3) contains supplementary material, which is available to authorized users.

## Background

Breast cancer is the most common cancer amongst women, both in developed and less developed countries. It is a global disease burden, accounting for approximately 1.67 million out of 14.1 million new cancer cases reported in 2012 [1]. Despite advancement and new discoveries that have revolutionised the management of breast cancer, it remains the leading cause of cancer-related deaths among women. In low- and middle-income countries (LMICs), incidence is lower than high-income countries, but has been rising by as much as five percent annually [1] because of the dramatic changes in social and lifestyle determinants, including changes in reproductive factors, environmental exposures, diet and exercise [2].

Although early detection of cancer improves survival, breast cancer screening remains controversial [3]. Mammographic screening has been well studied in high-income countries (HICs), especially among Caucasians and is shown to significantly reduce mortality. In most Western countries, population-based screening programmes are available with defined screening intervals and target populations [4]. By contrast, population-based screening is only available in four Asian countries, namely Japan, Korea, Singapore and Taiwan [5]. This is largely due to inadequate resources in other Asian countries to carry out national mammography programmes and lack of evidence on cost-effectiveness [6].

Breast cancer is the most common cancer in Malaysia, accounting for 18% of all cancers and 31% of cancers in women. Compared to women of European descent where the incidence peaks at 60–70 years [7], approximately half of the breast cancers detected in Malaysia were younger than 50 [8]. Notably, there is limited data on mammographic screening amongst younger women (40–49 years) particularly Asian women [9], and therefore, the age to initiate such screening among Malaysian women still remains controversial.

In Malaysia, like in the majority of Asian countries, a population-based mammographic screening programme is not feasible or cost-effective mainly because of the lower incidence and lack of resources. Breast cancer incidence in Malaysia is estimated to be 38.7 per 100,000 women, comparatively lower than Singapore (65.7 per 100,00) and higher income countries like the United Kingdom (95 per 100,000) [1]. Opportunistic mammographic screening in Malaysia is provided by both governmental agencies and non-governmental organisations (NGOs). However, there has been little information on the performance of such programmes, in terms of clinical follow-up and cancer detection rate.

The objective of this study is to describe the challenges encountered and to determine the cancer detection rate and adjunct ultrasonography recommendation rate of an opportunistic mammographic screening programme.

## Methods

### Recruitment

The Malaysian Mammography (MyMammo) study is a subsidised, opportunistic mammographic screening programme among Malaysian women aged 40 to 74 years in a private tertiary hospital that serves a suburban locality in Selangor, Malaysia. Selangor is home to 6.3 million people, 13% of which are women aged 40 to 74 years [10]. Screening was offered to women with no personal history of breast cancer who have not had a mammogram recently (at least a year prior to enrolment in the programme). Participants were recruited through flyers, posters and media. Written informed consent was obtained from all participants. Participants donated blood samples for research and completed a questionnaire (Additional file 1) that included demographic information, anthropometric data, menstrual and reproductive history, family history of cancer, and motivators and barriers for participating in the MyMammo Study.

From October 2011 to June 2015, a total of 1,966 subjects participated in the programme. Of these, 35 women have incomplete data and 153 women were symptomatic, leaving 1,778 women for analysis. All women had a clinical breast examination by a medical officer and were referred to a surgeon if there were any mammographic abnormalities requiring further assessment. Women with inconclusive and suspicious mammography reports (BIRADS categories 0, 3, 4, or 5) were called for further consultation and clinical examination with the surgeon. The reporting radiologists were radiologists without fellowship training in breast imaging.

The study protocol was developed in accordance with the principles of the Declaration of Helsinki and approved by the Sime Darby Medical Centre Independent Ethics Committee [201109.4].

### Mammographic screening and mammographic density measurements

Full Film Digital Mammography (FFDM) was performed using the Hologic Selenia system, with two views (mediolateral oblique and craniocaudal) for bilateral breasts. Mammograms were reported by trained radiologists. The recommendation and reasons for adjunct ultrasonography were stated in the mammography report. The findings were classified into 6 categories, based on the Breast Imaging Reporting and Data System (BIRADS) by the American College of Radiology (ACR).

The Volpara method was used to obtain volumetric mammographic density measurements, and was previously described [11]. In short, the Volpara software defines dense volume (cm^3^) as the integration of dense thickness at each pixel, across all pixels of the mammogram. Total breast volume (cm^3^) was calculated as a function of breast area and breast thickness. Percent dense volume (%) is the ratio of dense volume to total breast volume. The average percent dense volume of bilateral breasts in CC view was used as the measurement of breast density in this analysis.

### Statistical analysis

Descriptive statistics were used to describe the demographics of the cohort, as well as to describe the recall rate for adjunct ultrasonography and cancer detection rate of the mammographic screening programme. Univariate analyses, including independent-sample T-tests for continuous variables and chi-square tests of homogeneity for categorical variables, were used to determine factors associated with the recommendation for adjunct ultrasonography. As the sensitivity of mammography is dependent on mammographic density, it was incorporated into the analysis. Other factors that were included were age, menopausal status and parity as these factors are closely associated with breast density [12–14]. Logistic regression models were used to evaluate the effect of multiple variables on the recommendations for adjunct ultrasonography. Statistical tests were two-sided, and a *p*-value less than 0.05 was considered statistically significant. All statistical analyses were performed using IBM Statistical Package for Social Sciences (SPSS) version 23.0.

## Results

Between October 2011 and June 2015, a total of 1,778 asymptomatic women aged 40–74 years underwent subsidised mammographic screening. The majority of the participants were Chinese (65.4%), followed by Indians (16.5%), Malays (13.7%), and mixed or other ethnicities. Notably, Chinese women were more likely to attend screening and the demographics attending screening were different from that of the overall population where 24.6% of the population are Chinese, 7.3% are Indians, and 67.3% are Malays. Most of the women were well educated and from a relatively high socio-economic background. Ninety-three percent had at least secondary education, and 51.2% with a monthly household income of more than USD 1,200 (Table 1).

**Table 1:** Demographics of women enrolled in MyMammoStudy

Demographics	Range/Number of women	Mean/Percentage (%)
Age at enrolment (years)	40–74	50.75 (7.31)
Body-mass index (kg/m^2^)	14–47	24.96 (4.49)
Ethnicity		
Chinese	1163	65.4
Indian	293	16.5
Malay	244	13.7
Others	78	4.4
Education level		
Primary or less	123	6.9
Secondary	883	49.7
Tertiary	771	43.4
Missing data	1	0.1
Average monthly income (RM)		
≤ 5,000 [≤ USD 1,200]	817	46.0
5,000–10,000 [USD 1,200–2,300]	544	30.6
≥ 10,000 [≥ USD 2,300]	359	20.2
Missing data	58	3.3
Hormonal		
Age at menarche (years)		
< 12	220	12.4
12–13	1031	58.0
≥ 14	514	28.9
Unknown or data missing	13	0.7
Menopausal status		
Pre-/Peri-menopausal	920	51.7
Post-menopausal	857	48.2
Never had menses	1	0.1
Number of pregnancies		
0	237	13.3
1–2	534	30.0
3–4	737	41.5
≥ 5	268	15.1
Unknown or data missing	2	0.1
Age at first birth (years)		
< 20	63	3.5
20–24	340	19.1
25–29	678	38.1
≥ 30	452	25.4
Nulliparous	237	13.3
Unknown or missing data	8	0.4
Family history of breast cancer (1° relative)	260	14.6

### Cancer detection

Most (86.8%) of the screening mammograms were reported as BIRADS 1 and 2 (normal or benign findings), with 9.7% BIRADS 3 and 2.1% BIRADS 4 and 5 (suspicious or highly suggestive of malignancy).

Overall, Seven cancers or 0.39% [95% confidence interval: 0.10%, 0.68%] were detected. Six cancers were detected in women aged 50 and above with a cancer detection rate of 0.64% [0.13%, 1.15%]. Only one cancer was detected among women below 50, a detection rate of 0.12% [0%, 0.35%]. Of the seven cancers detected, one was ductal carcinoma in situ (Stage 0), three were Stage 1 and three were Stage 2.

### Adjunct ultrasonography

Recommendation for adjunct ultrasonography was made in 30.7% [28.6%, 32.8%] of women. The most common reason for recommendation was for regular nodularities and opacities, 41.2% [37.1%, 45.3%]. However, dense breasts alone accounted 20% [16.6%, 23.4%] of the recommendation for ultrasonography. Other reasons were for abnormalities such as breast asymmetry or micro-calcifications (Table 2).

**Table 2:** Reasons for adjunct ultrasonography recommendation

Reasons for recommendation	Number of cases (*n* = 546)	Percentage (%)
Regular nodularities or opacities	225	41.2
Dense breast with other abnormalities	205	37.5
Architectural distortion and asymmetrical densities	133	24.4
Dense breast only	111	20.3
Irregular/lobulated nodularities or opacities	91	16.7
Macro-calcifications (indeterminate)	65	11.9
Non-clustered micro-calcifications	48	8.8
Clustered micro-calcifications	30	5.5
Mammographically negative, but palpable lump	8	1.5
Spiculated/suspicious masses	5	0.9
History of breast lumps/cysts	5	0.9
Benign-looking masses	4	0.7
Family history of breast cancer	4	0.7

Factors affecting recommendation for adjunct ultrasonography were studied. Univariable analysis showed that younger women, premenopausal women and women with denser breasts were more likely to be recommended for adjunct ultrasonography. Radiologists who reported more than 360 mammograms (of the total of 1778 mammograms) were less likely to recommend adjunct ultrasonography compared to those who recommend less than 180 mammograms (Table 3). In multivariable analyses, only the interpretation volume by the radiologists remained significantly associated with ultrasonography recommendation (Table 4).

**Table 3:** Univariate analysis of variables associated with adjunct ultrasonography recommendation

Demographics	Adjuvant ultrasonography recommendation		
	No. of women (%)		*p*-value
	Recommended (*n* = 546)	Not recommended (*n* = 1,232)	
Age at enrolment (years)			
< 50	301 (55.1%)	545 (44.2%)	0.000*
≥ 50	245 (44.9%)	687 (55.8%)	
Mean age (SD)	49.93 (7.2)	51.12 (7.3)	0.002*
Body-mass index (kg/m^2^), mean (SD)	24.72 (4.8)	25.07 (4.3)	0.129
Ethinicity			
Chinese	360 (65.9%)	803 (65.2%)	0.719
Indian	95 (17.4%)	198 (16.1%)	
Malay	69 (12.6%)	175 (14.2%)	
Others	22 (4.0%)	56 (4.5%)	
Hormonal			
Age at menarche (years)			
< 12	76 (14.0%)	144 (11.8%)	0.082
12–13	295 (54.5%)	736 (60.1%)	
≥ 14	170 (31.4%)	344 (28.1%)	
Menopausal status			
Pre-/Peri-menopausal	320 (58.6%)	600 (48.7%)	0.001*
Post-menopausal	226 (41.4%)	631 (51.3%)	
Number of pregnancies			
0	78 (14.3%)	159 (12.9%)	0.094
1–2	180 (33.0%)	354 (28.8%)	
3–4	203 (37.2%)	534 (43.3%)	
≥ 5	85 (15.6%)	183 (14.9%)	
Age at first birth (years)			
< 20	23 (4.9%)	40 (3.7%)	0.021*
20–24	89 (19.1%)	251 (23.5%)	
25–29	195 (41.8%)	483 (45.3%)	
≥ 30	159 (34.1%)	293 (27.5%)	
Parity			
Parous	466 (85.3%)	1,067 (86.6%)	0.477
Non-parous	80 (14.7%)	165 (13.4%)	
Average volume density, (%)	14.02 (8.38)	12.69 (8.41)	0.036*
Radiologists			
Radiologist reporting < 180 mammograms	242 (44.3%)	176 (14.3%)	0.000*
Radiologist reporting > 360 mammograms	304 (55.7%)	1,056 (85.7%)	

*Statistically significant (*p*-value < 0.05)

**Table 4:** Multivariable analysis of factors associated with adjunct ultrasonography recommendation (categorical)

Variables	Adjunct ultrasonography recommendation	
	Odds radio; 95% CI	*p*-value
Age at enrolment (years)		
< 50	1.828 (1.275, 2.620)	0.001*
50 and above	1.00 (ref.)	
Body-mass index (kg/m^2^)	0.991 (0.952, 1.033)	0.678
Age at first birth (years)		
< 20	1.00 (ref.)	
20–24	0.497 (0.116, 2.128)	0.346
25–29	0.597 (0.148, 2.414)	0.469
≥ 30	0.703 (0.172, 2.880)	0.624
Volumetric Mammographic Density Measurement		
Average % volume density (Volpara)	1.017 (0.992, 1.041)	0.186
Radiologists		
Radiologist reporting < 180 mammograms	1.00 (ref.)	0.000*
Radiologist reporting > 360 mammograms	0.127 (0.089, 0.180)	

*Statistically significant (*p*-value < 0.05)

## Discussion

Our study of a subsidised opportunistic mammographic screening programme shows that this programme is able to provide equivalent cancer detection rates compared to population-based screening programmes. However, the low rate of cancer detection in women below 50 years and high recall rates for an adjunct ultrasonography would not make it cost-effective to screen women aged below 50.

The cancer detection rate of 0.39% demonstrated in this study is similar to the screening programmes in other Asian countries with similar incidence rates of breast cancer, 0.48% in Singapore [15] and 0.5% in Hong Kong [16]. It is also similar to a previous study on opportunistic mammographic screening in Malaysia, showing a cancer detection rate of 0.5% [17]. In contrast, the breast cancer detection rate in a high-income country, such as Switzerland, with a higher incidence rate of breast cancer was reported to be between 0.61% and 0.79% at the initial screen [18]. Notably, we report a low cancer detection rate of 0.12% in women below 50 years old, which is comparable to a similar study in Malaysia which reported 0.2% [17]. In contrast, a Japanese study showed a higher detection rate of 0.56% in women younger than 50 years, compared to 0.26% among women aged 50 and above [19]. Taken together, as the cancer detection rate is relatively low in this region, it may not be cost-effective to conduct a population-based mammographic screening programme [20].

Unlike population-based biennial screening programmes in Caucasian population where the adjunct ultrasonography rate is 2 to 4%, our study showed that 3 out of 10 women attending screening mammography were recommended for adjunct ultrasonography, which would correspond to a recall rate of 30% for additional procedures. International guidelines such the European guidelines recommend that less than 7% should be recalled for further assessment [4], as this would correspond to a sensitivity of 83.3%, which is considered an optimal trade-off between the benefit of finding additional cancers and the increased number of procedures for non-cancers and the associated anxieties experienced by women [21].

We report that one possible reason for the high recall rate is that in LMICs, there is a shortage of radiologists trained in breast radiology. As shown in this study, the higher the interpretation volume, the lower the recall rates for adjunct ultrasonography. Several studies have highlighted that recall rate is dependent on patient population, radiologists and systemic factors [21–23]. Radiologists who are less experienced in breast imaging lack confidence in passing a mammogram as normal [24]. The study by the Breast Cancer Surveillance Consortium (BCSC) group investigating the radiologists’ characteristics associated with interpretive performance in screening mammography in the United States concluded that fellowship training in breast imaging was the only characteristic significantly associated with improved sensitivity [25]. Of note, mammography is the only evidence-based screening modality for breast cancer and thus, much effort should be focused on maintaining and achieving high quality mammograms. More training courses to help radiologists continuously improve standards of practice and linking education programmes to individual radiologist’s performance could potentially reduce recall rate [26–30].

Another reason for the high recall rate is that the Asian women in our study may have mammographically denser breasts. They were more likely to be pre-menopausal, Asian women with lower BMI, which are all factors associated with denser breasts. A previous study in Malaysia showed a recall rate of 31.7% for ultrasonography, and was significantly higher among the younger cohort [17]. In our study, 20% of women were recommended for ultrasound for dense breasts alone, without any other abnormalities. Studies have demonstrated that Asian women have denser breasts compared to Caucasian women [31, 32].

Our subsided mammographic screening programme encountered several challenges. Compliance proved to be a challenge as around 11% of the women did not return to collect their mammogram reports. A third of the women had to be contacted several times especially when they were reported to have BIRADS 4 or BIRADS 5, requiring further assessment. Even when contacted, about 10% were reluctant to have further investigations, as they did not report any lumps. This clearly reflects the pivotal role of pre-screening counselling in enabling and ensuring that women understand the purpose and implications of mammography and the significance of follow-up. Compliance was highlighted as a challenge in an early detection programme by clinical breast examination in the Philippines where 42.4% of the women who were found to have a breast lump refused further investigations [33].

As the programme was conducted in a private centre and the majority of the women were not insured, many were referred to public hospitals for further investigations due to financial reasons. Although seven cancers were detected, we acknowledge the possibility of missing some cancers. The lack of an effective national cancer registry also hampered the process of tracing and identifying cancers that were missed or developed later. However since the cancer detection rate was similar to another opportunistic mammography screening programme conducted in a public hospital, we presume that there are very few, if any, missed cancers.

It is crucial to note than one out of the seven cancers detected refused any cancer treatment, opting for alternative therapy. Such phenomenon was also seen in Indonesia, in an early detection programme utilizing screening mammography and clinical breast examination, in which only 42.8% of the women diagnosed with breast cancer returned for treatment [34]. Hence, as well as identifying the challenges to screening, identifying barriers to treatment is essential to the success of any early cancer detection programme.

## Conclusion

A subsidised opportunistic mammographic screening programme funded by an NGO is able to provide equivalent cancer detection rates compared to other programmes reported in the region. The low detection rate in women below 50 years coupled with the high recall rates for an adjunct ultrasonography would not make it cost-effective to screen women aged below 50. To reduce the recall for an adjunct breast ultrasonography, it is imperative to have comprehensive and continuous training modules for radiologists, especially those without fellowship training in breast imaging. Undeniably, counselling plays a pivotal role in patient’s awareness, voluntary compliance and in improving patient care as a whole. Women should be counselled about the benefits and drawbacks of mammography screening, and the need for follow-up assessments for any suspicious findings. An early detection programme, such as this one, will have benefit in raising breast awareness among women.

## Additional file

Additional file 1: Mammogram Questionnaire. Malaysian 1000 Mammogram Study (MyMammoStudy). Patient questionnaire. (DOCX 568 kb)
