# Supplementary material for: Performance of a subsidised mammographic screening programme in Malaysia, a middle-income Asian country
Source: BMC Public Health. 2017 Jan 28;17:127. doi: 10.1186/s12889-017-4015-3 (PMC5273834; doi:10.1186/s12889-017-4015-3)
Supplement: Additional file 1: — Mammogram Questionnaire. Malaysian 1000 Mammogram Study (MyMammoStudy). Patient questionnaire. (DOCX 568 kb) [file 12889_2017_4015_MOESM1_ESM.docx]

STUDY RECRUITMENT INFORMATION

| Study Administration information | | | |
| --- | --- | --- | --- |
| Study Site | | | : |
| Doctor | | | : |
| Interviewed on | | | :       [dd/mm/yyyy] |
| Interviewed by | | | : |
| Study Subject’s Information [Affix Patient’s label if available] | | | |
| Name | | :        Female | |
| IC No. | | : | |
| DOB | | :       [dd/mm/yyyy] | |
| Nationality | | :  Malaysian | |
| Subject No | | : | |
| Consent date | | :       [dd/mm/yyyy] version | |
| Study Sample Collection Information | | | |
| Consent  Blood  Questionnaire  Mammogram | :  Yes, on: ___________ [dd/mm/yyyy]  No  Does patient want to know genetic test results?  Yes  No  :  Yes, on: ___________ [dd/mm/yyyy]  No  EDTA-Plasma  Plain-Serum  :  Yes, on: ___________ [dd/mm/yyyy]  No :  Yes, on: ___________ [dd/mm/yyyy]  No | | |

Name:

Date of Birth:

IC Number:

Address:

Telephone Number:

**I consent to participate in the above research study.**

I consent to give a blood sample which will be used for laboratory studies into the inheritance of breast cancer. This will include hormonal, cellular and genetic tests.

I agree to a member of the research team examining my hospital case notes, requesting copies of my mammograms and having access to my stored tumour material (if any) for laboratory tests.

I have read the accompanying information sheet, and I understand the purpose and the nature of the study. I understand that information obtained during this study will be securely stored on an electronic database and will not be used for any other purposes. I also understand that my participation in this study is entirely voluntary and that I may withdraw from any or all parts of it, at any time, without giving a reason and without affecting my further care.

­­­­­­­­­­­­­­_________________________________ ______________________ _______________

*Name of Participant Signature of Participant Date*

­­­­­­­­­­­­­­_________________________________ ______________________ _______________

*Name of Person Obtaining Consent Signature Date*

­­­­­­­­­­­­­­_________________________________ ______________________ _______________

*Name of Witness Signature Date*

*(Required only if informed consent is requested orally from the participant)*

Thank you for agreeing to participate in this study. We are most grateful for your help.

Your answers will be treated in the strictest confidence and will not be disclosed to any third party, including your doctors. The information you provide will be used only for statistical analysis. It will not be possible to identify individuals in any results.

Just put a tick in the box under the appropriate answer, like this

OR write a number in the box, like this **1 2 3**

OR write details on a line, like this **Sime Darby Medical Centre**

If you do not know the answer to a question, or cannot remember please put “**NK**” (meaning **N**ot **K**nown).

If a question does not apply to you please put “**NA**” (meaning **N**ot **A**pplicable). If we have not given you enough room for some of your answers, please use a blank sheet.

**Date of completion:** //

**Contact address/telephone:**

Prof. Dr. Teo Soo Hwang, Ms. Yoon Sook-Yee, Ms. Shivaani Mariapun,

Cancer Research Initiatives Foundation, Sime Darby Medical Centre,

1 Jalan SS12/1A, Subang Jaya, 47500 Selangor, MALAYSIA

Telephone: 03 5639 1874

**Please answer the following questions before you proceed to the next page:**

I. How did you find out about the My1000Mammo study? *(You may tick more than one box)*

Radio or Newspaper

Posters (SDMC/ CARIF event)

E-mail from friend or family

Personal recommendation by friend or family

Recommendation by doctor or other staff at SDMC/ other hospital

Other, please specify: ………………………………………

II. What motivated you to take part in the My1000Mammo study? *(You may tick more than one box)*

I am worried about breast cancer

I was referred by my doctor / health screening centre

I was persuaded by a friend or a family member

I want to contribute to breast cancer research

I am eligible for a RM50 mammogram when I take part

Other, please specify: ………………………………………

**GENERAL INFORMATION ABOUT YOURSELF**

1 What is your **full** name? ............................................................................

What was your maiden name (*if applicable*) ............................................................................

2 What is your IC number?

3 What is your address? ............................................................................

………………………………………………………………………...

4 What is your telephone number? -

What is your mobile phone number? -

5 What is your date of birth (dd/mm/yyyy)? //

6 What is your height (in cm or in feet and inches)? . cm **OR**  ft  in

7 What is your current weight (in kg or in pounds)? . kg **OR** . lb

8 Which picture best describes the way you looked at age 7 years, age 18 years and now*.*


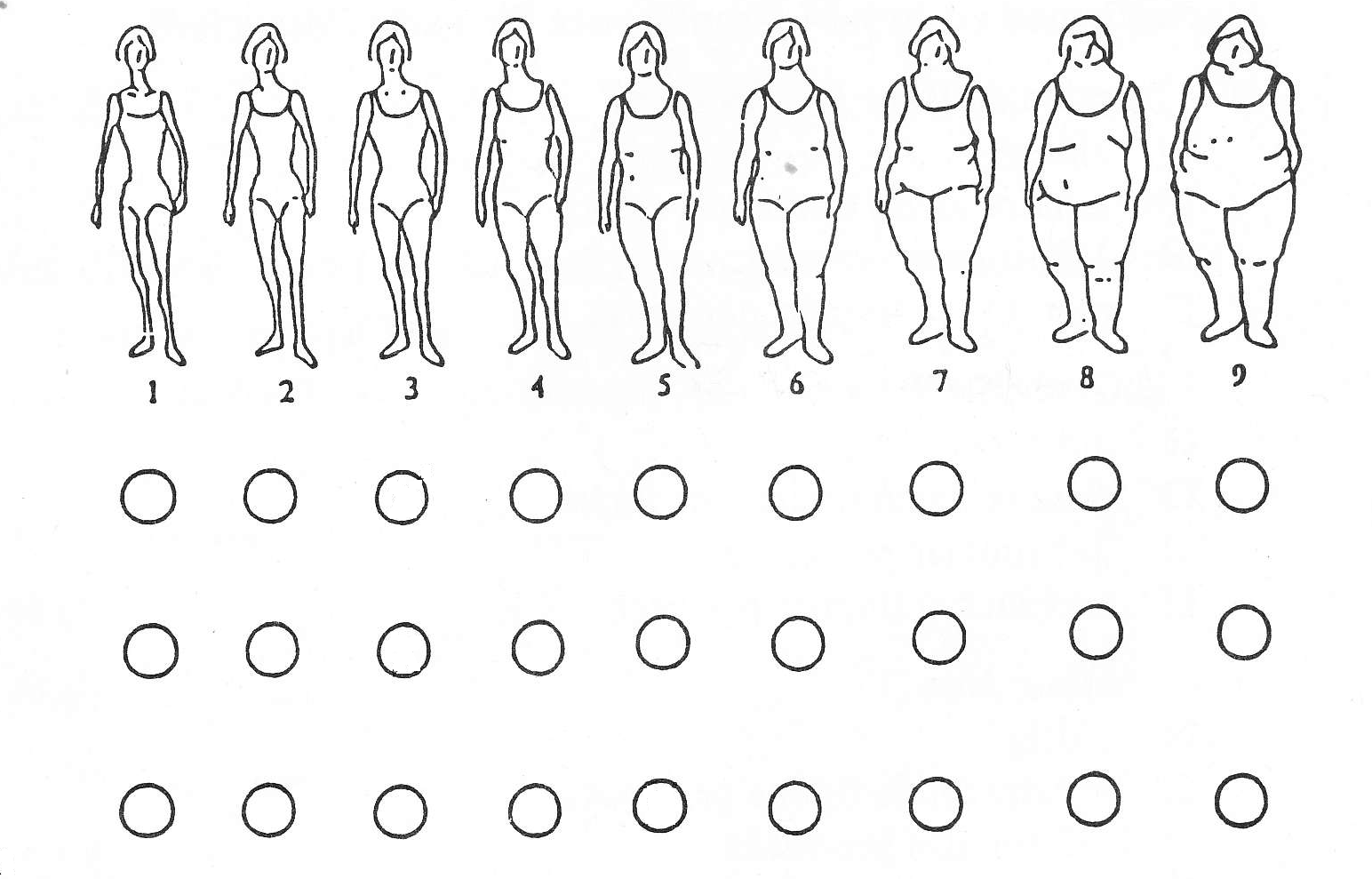


| 18 years  Now  7 years | **Cannot Recall** |
| --- | --- |

9 How would you describe your ethnic origin?

Malay  Chinese  Indian  Other, please specify

10 What is your highest education level?

Primary  Secondary  University  Other, please specify

11 What is your average monthly household income?

<5,000  5-10,000  10-50,000  >50,000

**INFORMATION ABOUT YOUR HEALTH**

12 How much physical activity/sports do you practice? *(Please indicate how much of each type of exercise you practice/practiced)*

**Strenuous Exercise:**

which normally makes your heart beat rapidly AND leaves you breathless, e.g. jogging, vigorous swimming or cycling, aerobics

**Moderate Exercise:**

which normally leaves you exhausted but not breathless, e.g. brisk walking, dancing, easy swimming or cycling, badminton, sailing, yoga, pilates, taiqi. qigong

**Gentle Exercise:**

which normally leaves you tired but not exhausted, e.g. walking, driving, housework (including

washing windows and polishing), gardening, DIY, golf.

| Childhood (before 18-years of age)   \| **Strenuous Exercise** \| **Moderate Exercise** \| **Gentle Exercise** \| \| --- \| --- \| --- \| \| Never  Less than 1 hour per week  1-2 hour per week  More than 2 hours per week \| Never  Less than 1 hour per week  1-2 hour per week  More than 2 hours per week \| Never  Less than 1 hour per week  1-2 hour per week  More than 2 hours per week \| |
| --- | --- | --- | --- | --- | --- | --- |
| 18-30-years of age   \| **Strenuous Exercise** \| **Moderate Exercise** \| **Gentle Exercise** \| \| --- \| --- \| --- \| \| Never  Less than 1 hour per week  1-2 hour per week  More than 2 hours per week \| Never  Less than 1 hour per week  1-2 hour per week  More than 2 hours per week \| Never  Less than 1 hour per week  1-2 hour per week  More than 2 hours per week \| |
| The most recent years   \| **Strenuous Exercise** \| **Moderate Exercise** \| **Gentle Exercise** \| \| --- \| --- \| --- \| \| Never  Less than 1 hour per week  1-2 hour per week  More than 2 hours per week \| Never  Less than 1 hour per week  1-2 hour per week  More than 2 hours per week \| Never  Less than 1 hour per week  1-2 hour per week  More than 2 hours per week \| |

**INFORMATION ABOUT YOUR HEALTH**

13 Have you ever smoked cigarettes?  Yes  No

**If yes**, do you still smoke?  Yes  No

For how many years have you ever smoked?  Years

| How much did you smoke on average during different periods of your life? | | | | | |
| --- | --- | --- | --- | --- | --- |
| Age | Number of cigarettes/day | | | | |
|  | 0 | 1-5 | 6-10 | 11-20 | >20 |
| Before age 20 years |  |  |  |  |  |
| 20-29 years |  |  |  |  |  |
| 30-39 years |  |  |  |  |  |
| 40-49 years |  |  |  |  |  |
| 50-59 years |  |  |  |  |  |
| 60 year or more |  |  |  |  |  |
| 1 year prior to your cancer diagnosis |  |  |  |  |  |

14 Have you ever drunk alcohol more than once a month on average?  Yes  No

(Alcohol includes beer, wine, hard liquor)

**If yes**, how much do you drink on average?

1 glass per day  1 glass per week  1 glass per month  Other, please specify

15 Do you drink **coffee** regularly?  Yes  No

**If yes**, at what **age** did you start to drink coffee regularly?  Years

**If yes**, how much do you drink on average?

1 cup per week  1 cup per day  1-5 cups per day  > 5 cups per day

16 Do you drink **tea** regularly?  Yes  No

**If yes**, at what **age** did you start to drink tea regularly?  Years

**If yes**, what type of tea do you drink regularly?

Black tea  Green tea  Herbal tea  Other, please specify

And, how much do you drink on average?

1 cup per week  1 cup per day  1-5 cups per day  > 5 cups per day

17 Do you drink **soya** **bean** milk regularly?  Yes  No

**If yes**, how much do you drink on average?

1 cup per week  1 cup per day  1-5 cups per day  > 5 cups per day

18 Do you eat soy products (tofu, tofufah, tempe, tau kua) regularly?

Every meal  Every day  Once a week  Other, please specify

19 Have you ever been told by a doctor that you have diabetes?  Yes  No

**If yes**, are you **currently taking** any medication for your diabetes?  Yes  No

**If yes**, please give the name(s) of the medication(s) you are **currently taking** for diabetes:

……………………………………………………………………………………………………………………………………………………...

**INFORMATION ABOUT MENSTRUATION, PREGNANCY AND CHILDBIRTH**

20 How old were you when your periods began?  Years

21 Are you still having periods?  Yes  No

**If yes**, are they regular or irregular?  Regular  Irregular

22 If you are no longer having periods, how old were you when they stopped completely?

Years

**And**, why and when (dd/mm/yyyy) did they stop?

It stopped by itself //

Uterus was removed //

Ovaries were removed //

Hormone treatment was stopped //

Other reason …………………………………………………………………………

Don’t recall

23 Have you ever been pregnant?  Yes  No

| If Yes, please state all pregnancies (including miscarriages and still births), birth year and birth weight for all children you gave birth to and number of months you breast fed each child. | | | | | | | |
| --- | --- | --- | --- | --- | --- | --- | --- |
| Pregnancy | Child/ Stillborn/  Miscarriage | Birthyear | | Birthweight (g) | Don’t recall | Breastfeeding  (months) | Don’t recall |
| 1 |  |  |  | |  |  |  |
| 2 |  |  |  | |  |  |  |
| 3 |  |  |  | |  |  |  |
| 4 |  |  |  | |  |  |  |
| 5 |  |  |  | |  |  |  |
| 6 |  |  |  | |  |  |  |
| 7 |  |  |  | |  |  |  |
| 8 |  |  |  | |  |  |  |

24 Have you ever been treated for infertility?  Yes  No

**If yes**, what treatment did you receive and for how long? ……………………………………………………..

25 Have you ever used the contraceptive pill?  Yes  No

**If yes**, for how long, in total? ……………………………………………………..

**And** are you currently taking the contraceptive pill?  Yes  No

26 Have you ever used hormone replacement therapy (HRT)?  Yes  No

**If yes**, for how long in total? ……………………………………………………..

**And**, are you currently using hormone replacement therapy?  Yes  No

**INFORMATION ABOUT MAMMOGRAMS**

27 Why are you coming for a mammogram today? *(You may tick more than one box)*

I am at the right age for screening

Someone in my family has/had cancer

I have a symptom (e.g lump, nipple discharge, etc.)

I had a lump/other symptoms previously

My friend/family member asked me to come

My doctor advised me to come

Other, please specify

28 Have you ever had a mammogram?  Yes  No

**If yes**, please answer question **29**

**If no**, please answer question **30**

29 When did you have your **first** mammogram?

(*Please tell us your age or the year (dd/mm/yyyy))*  Years **OR** //

Have you ever had a mammogram in this hospital?  Yes  No

Who advised you to have your **first** mammogram?

Myself

Doctor: General practitioner (GP)/ Breast surgeon/ Gynaecologist/ Other doctor

Family / Friend: Father/ Mother/ Sister/ Brother/ Daughter/ Son/ Relative/ Friend

Public campaign: Breast Cancer Awareness Campaign/ Advertisement/ Other

Other: ………………………………………………………………………………………………………………

When and where did you have your **most recent** mammogram? //

Name of screening unit/hospital ……………………………………………

Excluding this mammogram, how many mammograms have you had?

**And**, approximately how often do you have a mammogram? ……………………………………………

Was any abnormality ever detected?

**If yes**, please give details ……………………………………………

30 If you have **not** had a mammogram before, why? *(You may tick more than one box)*

I don’t think that I am at risk (too young/ no family history/other reasons)

I am worried that it may be painful

It is too expensive

I don’t want to know

Other, please specify

Who advised you to have this mammogram?

Myself

Doctor: General practitioner (GP)/ Breast surgeon/ Gynaecologist/ Other doctor

Family / Friend: Father/ Mother/ Sister/ Brother/ Daughter/ Son/ Relative/ Friend

Public campaign: Breast Cancer Awareness Campaign/ Advertisement/ Other

Other: ………………………………………………………………………………………………………………

**INFORMATION ABOUT YOUR HEALTH**

31 Have you ever had surgery for a benign lump or cyst in the breast?  Yes  No

32 Have you ever been diagnosed with breast cancer?  Yes  No

33 Have you ever been diagnosed with any other type of cancer?  Yes  No

**If yes,** what type? ………………………………..

How old were you when you were first diagnosed with this cancer?  Years

34 Have you ever had a gynaecological surgery?  Yes  No

If yes, what type and in what year? //

Sterilisation (e.g tubal ligation)

Removal of one ovary (oophorectomy)

Removal of both ovaries (oophorectomy)

Removal of uterus (hysterectomy)

Removal of uterus and part of cervix (hysterectomy)

Removal of uterus, cervix, ovaries and fallopian tube (total hysterectomy/TAHBSO)

Other, please specify: …………………………………………...

**FAMILY HISTORY**

We would like to ask you some questions about your **blood relatives**. Please only include details about your natural parents, children who are genetically related to you (not adopted children or step-children) and full brothers and sisters. We will ask you about any half brothers and sisters later in the questionnaire. We ask for this information because we are trying to understand about cancer in families. **Please answer all questions.**

**35 INFORMATION ABOUT YOUR MOTHER**

What is/was your mother’s **full** name? …………………………………………………………………

What was her maiden name? …………………………………………………………………

How would you describe her ethnicity?

Malay  Chinese  Indian  Other, please specify

Her most recent town of residence

*(or if deceased, last town and county of residence)*  …………………………………………………………………

What was her date of birth? (dd/mm/yyyy) //

Is she still alive?  Yes  No

**If no,** what was her date of death? //

Did she ever have cancer?  Yes  No

**If yes***,* what type of cancer? …………………………………………………………………

**And** how old was she when it was diagnosed?  Years

**36 INFORMATION ABOUT YOUR FATHER**

What is/was your father’s full name? …………………………………………………………………

How would you describe his ethnicity?

Malay  Chinese  Indian  Other, please specify

His most recent town of residence

*(or if deceased, last town and county of residence)* …………………………………………………………………

What was his date of birth? //

Is he still alive?  Yes  No

**If no,** what was his date of death? //

Did he ever have cancer?  Yes  No

**If yes***,* what type of cancer? …………………………………………………………………

**And** how old was he when it was diagnosed?  Years

**37 INFORMATION ABOUT YOUR BROTHERS AND SISTERS**

How many full brothers and sisters do you have/have you had?

| **Full name** | **Date of Birth**  **(day/month/yr)** | **Sex** | **Did he/she ever have cancer?** | **If yes, what type of cancer?** | **Age when diagnosed?** | **Is he/she still alive?** | **If not, what was their date of death?** | **Town of residence** |
| --- | --- | --- | --- | --- | --- | --- | --- | --- |
|  |  |  |  |  |  |  |  |  |
|  |  |  |  |  |  |  |  |  |
|  |  |  |  |  |  |  |  |  |
|  |  |  |  |  |  |  |  |  |
|  |  |  |  |  |  |  |  |  |
|  |  |  |  |  |  |  |  |  |
|  |  |  |  |  |  |  |  |  |
|  |  |  |  |  |  |  |  |  |
|  |  |  |  |  |  |  |  |  |
|  |  |  |  |  |  |  |  |  |
|  |  |  |  |  |  |  |  |  |
|  |  |  |  |  |  |  |  |  |
|  |  |  |  |  |  |  |  |  |
|  |  |  |  |  |  |  |  |  |

**38 INFORMATION ABOUT YOUR CHILDREN**

How many children do you have/have you had?

| **Full name** | **Date of Birth**  **(day/month/yr)** | **Sex** | **Did he/she ever have cancer?** | **If yes, what type of cancer?** | **Age when diagnosed?** | **Is he/she still alive?** | **If not, what was their date of death?** | **Town of residence** |
| --- | --- | --- | --- | --- | --- | --- | --- | --- |
|  |  |  |  |  |  |  |  |  |
|  |  |  |  |  |  |  |  |  |
|  |  |  |  |  |  |  |  |  |
|  |  |  |  |  |  |  |  |  |
|  |  |  |  |  |  |  |  |  |
|  |  |  |  |  |  |  |  |  |
|  |  |  |  |  |  |  |  |  |
|  |  |  |  |  |  |  |  |  |
|  |  |  |  |  |  |  |  |  |
|  |  |  |  |  |  |  |  |  |
|  |  |  |  |  |  |  |  |  |
|  |  |  |  |  |  |  |  |  |

**39 HAVE ANY OF YOUR OTHER BLOOD RELATIVES BEEN DIAGNOSED WITH CANCER?**  Yes  No

*(e.g. aunt, uncle, grandmother, grandfather, first cousin)*

How many full **brothers** does your **mother** have/has your mother had?

How many full **sisters** does your **mother** have/has your mother had?

How many full **brothers** does your **father** have/has your father had?

How many full **sisters** does your **father** have/has your father had?

| **Relationship (please do not give their name)** | **Maternal (mother’s side)/ Paternal (father’s side)** | **What type of cancer?** | **Age when diagnosed?** | **Is he/she still alive?** | **If not, what was their age at death?** | **And, what was their year at death?** |
| --- | --- | --- | --- | --- | --- | --- |
|  |  |  |  |  |  |  |
|  |  |  |  |  |  |  |
|  |  |  |  |  |  |  |
|  |  |  |  |  |  |  |
|  |  |  |  |  |  |  |
|  |  |  |  |  |  |  |
|  |  |  |  |  |  |  |
|  |  |  |  |  |  |  |
|  |  |  |  |  |  |  |
|  |  |  |  |  |  |  |

Thank you for taking the time to complete this questionnaire. If there were any questions you did not understand, or if you have any questions about the study, please do not hesitate to contact one of the study organisers whose names and contact details are given on the inside cover of the questionnaire.

If we have any queries about the answers you have given in this questionnaire it would be helpful if we could phone you to clarify them. If however, you do not wish us to contact you, please say so.

You **may*/may not*** contact me to clarify any of my answers which you are not clear about. (*please delete as appropriate)

Thank you once again. We are very grateful for your help. Would you now please sign and date this questionnaire.

Signed ............................................................ Date ...................................
